# Retrospective Review of Outcomes Related to Early Therapy Intervention Following Application of Cultured Epidermal Autografts in Severely Burned Patients

**DOI:** 10.1093/jbcr/irae060

**Published:** 2024-04-11

**Authors:** Sarah Sabbatini, Sai R Velamuri, David M Hill

**Affiliations:** Burn Rehabilitation Department, Regional One Health, Memphis, TN 38103, USA; Department of Surgery, University of Tennessee Health Science Center, Memphis, TN 38103, USA; Department of Pharmacy, Regional One Health, Memphis, TN 38103, USA

**Keywords:** cultured epidermal autografts, burns, early ambulation, aftercare, rehabilitation

## Abstract

Cultured epidermal autografts (CEA) have since become more prevalent in the treatment of burn-injured patients with limited available donor sites for adequate wound closure, resulting in decreased mortality rates and an increased number of these patients requiring burn therapy services to achieve optimal functional outcomes at discharge. However, the use and postoperative management of CEA continue to be controversial due large to the physiological fragility and expense of CEA, leading to variable postoperative treatment practices across burn centers. As such, minimal research is available regarding patient outcomes following CEA application, specifically related to burn therapy intervention. Thus, a retrospective chart review was conducted on a series of 10 patients, 18 years of age or older, admitted to a single, American Burn Association verified burn center, between April 2015 and April 2023, who required CEA and received pre- and postoperative treatment by burn therapists in accordance with center-specific burn rehabilitation guidelines. The resulting patient outcomes, in response to early implementation of therapy interventions post-CEA surgery, demonstrated optimal functional status for patients upon discharge and positive long-term implications.

## INTRODUCTION

Since the first clinical application in 1981 by O’Connor et al., the use of cultured epidermal autografts (CEA) has been proven an effective complement to split-thickness skin grafts in successful wound closure of patients with large burn wounds, covering more than 30% of the total body surface area (TBSA).^1,2^ In recent years, the use of CEA by burn surgeons has become more prevalent in the treatment of patients with large TBSA and limited donor sites available for adequate wound closure.^3^ As surgical application of CEA becomes more widespread and as burn care continues to advance, the mortality rate of severely burn-injured patients has steadily decreased, resulting in an increased number of critically ill burn patients requiring rehabilitation services if positive functional outcomes are to be achieved by discharge.^4^

In addition to high mortality risk, severe burn injury is associated with a multitude of physiological challenges (ie, muscle wasting, compromised cardiovascular endurance, scar tissue formation, potential for skin and joint contractures, etc.), all of which can lead to poor functional outcomes. Studies support the practice that burn patients who receive early mobilization training in the acute stages of their intensive care unit (ICU) stay have better overall functional outcomes compared to those who do not.^5,6^ Although it is becoming more commonplace for therapy protocols to focus on early rehabilitation of patients in the burn ICU, limited research exists regarding burn therapy intervention and outcomes in patients following surgical application of CEA. The lack of research is most likely due to the fact that postoperative treatment of patients following CEA surgery varies greatly across burn centers, depending on burn team experience and perceptions of CEA fragility and expense. The objective of this retrospective review was to assess one burn center’s functional outcomes at discharge in CEA-treated patients as a result of early intervention by burn therapists (PT/OT) educated in pre- and postoperative management of CEA through center-specific burn rehabilitation guidelines.

## METHODS

### Study design and study population

This retrospective case study received dual Institutional Review Board (IRB # 22-09140-XP) approval and included patients admitted to a single, American Burn Association (ABA) verified burn center between April 2015 and April 2023. Patients were eligible for the study if they were 18 years of age or older, underwent surgical application of CEA during their stay, and received treatment by burn therapists.

### Data collection

Patients were identified through retrospective electronic health record review. Demographics, injury characteristics, and surgical procedure data were collected, including age, gender, ethnicity, percent TBSA burned, mechanism of injury, location and depth of burn wounds, number of surgical procedures, location of CEA, percent CEA-graft take, and interventions and treatment plans by burn therapists.

### Statistical analysis

The study timeframe was chosen based on expected data availability. A sample size calculation was not performed due to the expected rarity of the CEA application. Dichotomous data were presented as numbers and percentages. Continuous data were visually inspected and reported as either mean with standard deviation or median with the interquartile range, depending on approximate distribution and skew. All data was collected and analyzed in Microsoft Excel 2019 (Version 2304).

### Burn rehabilitation guidelines

The burn rehabilitation guidelines utilized in this study were designed by a dedicated burn therapy team made up of highly trained and educated physical and occupational therapists with over 40+ years of experience in treating burn patients following the application of CEA. These guidelines were established to maximize functional outcomes in patients who require CEA surgery for burn wound closure and to combat the negative effects of prolonged immobility that contribute to functional impairment.^5^

### Pre-CEA (within 24-72 h of admission)

As soon as possible following a patient’s admission to the burn center (eg, within the first 24-72 h): (1) the on-call burn surgeon will identify the patient as a potential candidate for the use of CEA, (2) physical therapy and occupational therapy evaluations are completed, and (3) and in most cases, the patient is taken to the operating room (OR) for wound bed preparation through burn wound excision with the placement of allograft or skin substitute, and harvesting of a full-thickness skin biopsy for CEA growth.^2^

### Pre-CEA (3- to 4-week period)

During the approximate 3- to 4-week period it takes for the keratinocytes derived from the full-thickness biopsy to grow and then be delivered as sheets stapled to petrolatum gauze backings, patients will receive daily hydrotherapy and burn wound assessment to decrease the risk for infection.^2^ If the patient is being treated pre-CEA with allograft coverage, the patient will return to the OR approximately once a week for allograft removal, potential re-excision, and wound bed assessment.^2,7^ Patients being treated with a skin substitute for burn wound coverage during the CEA-growth period will be closely monitored and assessed daily in hydrotherapy to ensure the wound bed remains free of infection. When the surgeon has determined that the dermal layer of the skin substrate utilized has provided a viable wound bed for the CEA, the patient is taken back to the OR a final time for CEA application. During surgery, the allo-epidermis is excised and removed, leaving the allo-dermis as the base layer for the CEA. CEA sheets are then applied over a widely meshed split-thickness autograft.^2^ Alternatively, if a skin substitute has been used, the skin substitute is removed, the wound bed is excised to healthy tissue, and again, the CEA sheets are applied in combination with a wide meshed split-thickness autograft. Ideally, the dermal components used as the base layer for CEA application will allow for the CEA to differentiate into normal dermis so that epidermal regeneration can occur.^2^

Therapy intervention during this time is extremely important, specifically as it relates to achieving and maintaining adequate and functional joint motion. Prior to the CEA application, patients are treated up to 3-5 h per day by burn therapists, 5-7 days per week. Treatment during this time includes but is not limited to, a variety of elongation techniques, out-of-bed functional mobility training, and strength and endurance training. Additionally, patients are placed on a strict positioning regimen that is collaboratively deployed by all burn staff, especially nurses, for the continuity of the patient’s care. Positioning not only promotes day-to-day carryover in motion from a patient’s therapy sessions but also helps to decrease the potential for contracture formation and any unwanted skin breakdown unrelated to a patient’s burn wounds.

The education of the patient and any family members or caregivers involved is also an important aspect of the patient’s care, and it should be initiated as early as possible following admission. A good understanding of the overall plan of care by the patient and their support system can potentially facilitate positive long-term outcomes.

### Post-CEA (postoperative day [POD] 0-2)

Immediately following a patient’s CEA surgery, burn therapists are present upon a patient’s return to their ICU room to begin proper positioning intervention for minimizing unwanted pressure to areas where CEA was just applied. Typically, if the CEA-treated sites include one or more of the patient’s limbs, positioning is conducted through the use of specific positioning devices made from thermoplastic pipe and large-sized elasticated tubular dressings meant to elevate the targeted area through suspension of the limb(s) without causing increased pressure to the grafts. Additionally, if a patient’s positioning regimen prior to CEA application incorporated the use of custom orthoses or splints to CEA-treated areas, these positioning devices are not utilized again until after CEA-graft take is assessed upon initial takedown of the CEA backings (approximately POD 7-10). Furthermore, education materials detailing the patient’s specialized positioning regimen and individualized wound care schedule are provided by the therapists to all burn staff involved in the patient’s care and are visually displayed inside and outside of the patient’s room in accordance with facility protocols.

Burn therapists are also closely involved in a patient’s transfers and wound care during this time period. If a patient is to be transferred in any capacity within the first 48 h of CEA application, the therapists will work closely with the nursing staff to ensure that all transfers are performed appropriately, with careful attention to high-risk areas susceptible to shear forces and increased pressure. As previously mentioned, therapists assist in the complex wound care and dressing changes that occur during this timeframe to ensure all areas of the body where CEA are present remain stabilized and are handled appropriately, including removal and reapplication of dressings around the patient’s bilirubin light schedule. Bilirubin lights are used postoperatively in CEA-treated patients for approximately 7-10 days to reduce potential opportunistic infections and to facilitate the healing of CEA by maintaining a dry environment. During a 24-h period, a patient’s CEA sites will alternate between bilirubin light therapy with no compression or gauze dressings present (“drying phase”; typically, 8-9 h at a time), and donning of silver nitrate-soaked gauze dressings under lightly applied compression wraps (typically, 3-4 h at a time).

### Post-CEA (POD 2-7)

Beginning about POD 2-7, elongation techniques are reinitiated with careful attention to hand placement and visual assessment of the CEA response to movement of surrounding soft tissue and joint structures. The goals of this period are to regain functional joint motion in preparation for out-of-bed mobilization and to counteract scar tissue formation in an attempt to decrease the potential for joint or skin contractures to occur. It is preferred that initial elongation sessions occur with at least 2 burn therapists’ involvement: one therapist present to perform the passive elongation techniques to the surrounding soft tissue and targeted joints, and one therapist present to provide stabilization of the area being treated, ensuring all CEA sites are protected from unwanted movement or shear forces. Elongation is typically conducted during the last 1-2 h of a patient’s bilirubin light treatment, in the absence of outer burn dressing layers, so that the therapists have clear visibility of CEA sites during elongation intervention. This timeframe also allows the therapists to be present and assist with the reapplication of burn dressings upon the conclusion of elongation treatment.

### Post-CEA (POD 7+)

About 1 week after CEA surgery, and upon physician approval, burn therapists can begin mobilizing the patient at the edge of the bed or out of bed, depending on the patient’s medical and functional status. This timeframe can also vary depending on the location of the CEA, related to weight-bearing surfaces, and the patient’s ability to participate and understand a therapist’s instruction appropriately.^8^ When the patient is deemed appropriate to begin functional mobility training, these sessions will occur opposite the patient’s bilirubin light therapy, during the phase in which a patient dons shear-avoidant dressings to decrease the potential of shear forces causing harm to CEA sites during mobility. Shear-avoidant dressings include a layer of bridal veil over the contact dressing, then a layer of dry gauze, followed by carefully applied compression wraps with underlying foam sheets to certain areas for additional protection of CEA sites and bony prominences. If necessary, burn therapists can also resume orthotic and splinting interventions during this time period.

### Post-CEA (POD 10+)

Treatment in the burn therapy clinic can resume as early as POD 10, given there have been no complications up to this point. During this phase, burn therapists will begin advancing the intensity, frequency, and duration of a patient’s treatment program to include, but not limited to, advanced functional mobility and gait training, total body strengthening and resistance training, muscular endurance training, and patient and family education, in preparation for the patient’s discharge home or transition to next level of care (ie, burn-specific inpatient rehab facility).

## RESULTS

Demographics, injury characteristics, and surgical data of the 10 included patients can be found in Table 1. The average age was 42.5 ± 9.2 years, and most were Caucasian males. All of the patients sustained thermal burn injuries, with 90% involving the head, neck, and lower limbs. The average TBSA burned was 71% ± 16.4%, categorizing all 10 patients as severely burned per ABA guidelines.

**Table 1.** Demographics, Injury Characteristics, Surgical Procedure Data

Total number of study patients	10
Age, year^a^	42.5 ± 9.2
Ethnicity, *n* (%)	
Caucasian	8 (80)
African American	2 (20)
Gender, *n* (%)	
Male	9 (90)
Female	1 (10)
TBSA burns, %^a^	71 ± 16.4
Mechanism of injury, *n* (%)	
Thermal	10 (100)
Location of burn, *n* (%)^b^	
Head and neck	9 (90)
Trunk	10 (10)
Upper limb(s)	10 (10)
Lower limb(s)	9 (90)
Total number of operations^a^	14 ± 7.2
Total number of CEA operations^a^	1.5 ± 0.7
Location of CEA, *n* (%)^b^	
Head and neck	2 (20)
Trunk	8 (80)
Upper limb(s)	7 (70)
Lower limb(s)	8 (80)

^a^Mean ± standard deviation.

^b^Total will add to more than 100%, as multiple locations could be burned on each patient.

Abbreviations: CEA, cultured epidermal autografts; TBSA, total body surface area.

**Figure 1.**
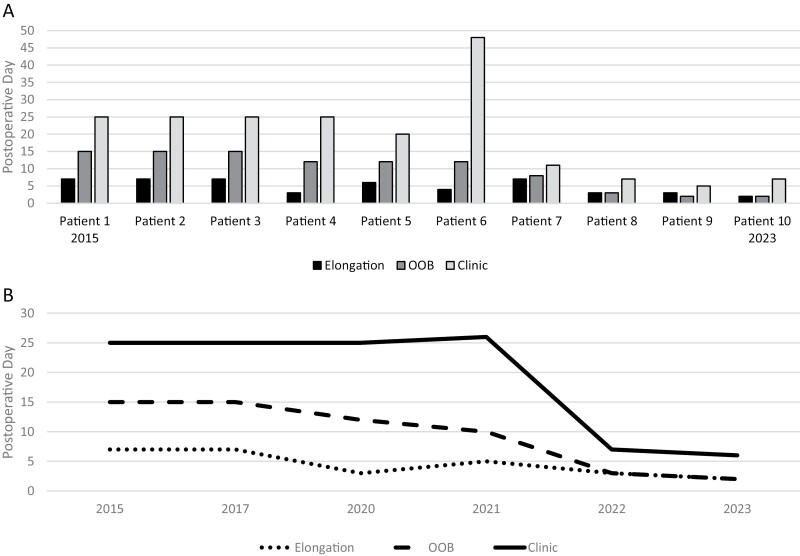
Initiation of Elongation Intervention, Out-of-Bed Mobilization (OOB), and Resumption of Treatment in Burn Therapy Clinic (Clinic) by Postoperative Day. (A) Demonstrates Postoperative Day of Initiation of Each Intervention by Study Patient (Patients Presented Chronologically by Admission Date). Note, Patient 6 on Contact Precaution Orders Restricts the Patient From Being Treated Outside of the Burn ICU Room. With This Exception, There Was a Notable Decrease in Time (Days) with the Initiation of Each Intervention From Patient 1 to Patient 10. (B) Demonstrates Initiation of Each Intervention Over Time, Using the Average of Values According to Patients’ Year of Injury. There Was a Clear Trend in the Earlier Time to Initiation of Each Intervention From 2015 to 2023

**Figure 2.**
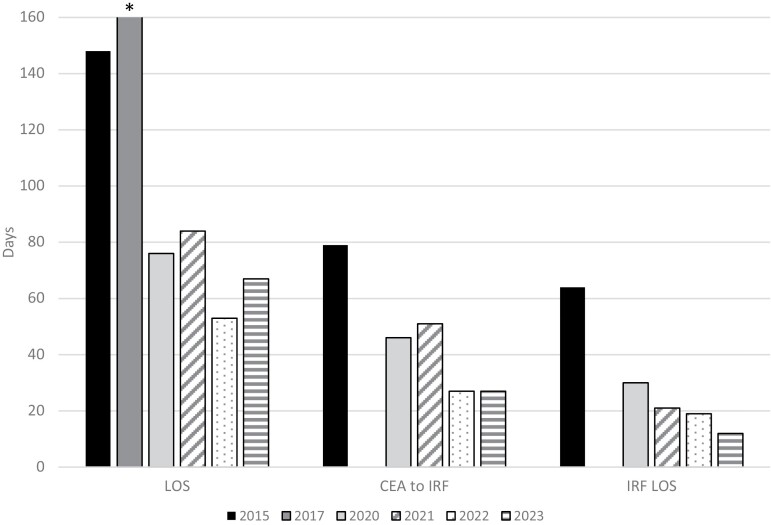
Evolution of Outcomes Over Time. Note, There Was Not a Patient Admitted for Each Year of the Time Period, So Each Year Is Not Represented; However, It Is Presented Chronologically and as an Average of Values According to Patients’ Year of Injury. LOS, Days From Final Cultured Epidermal Autograft (CEA) Operation to Burn-Specific IRF Admission (CEA to IRF), and LOS in IRF (IRF LOS) All Decreased Over Time. *The One Patient Admitted in 2017 Had an Extremely Skewed LOS Due to Complications and Social Barriers Unrelated to CEA Placement. As Such, the Patient’s Depicted LOS Was Truncated to Better Depict the Greater Whole. Related, This Patient Was Not Admitted to IRF; Therefore, No Data Was Presented for 2017 for CEA to IRF or IRF LOS

The median day of resumed therapy interventions post-CEA surgery is displayed in Table 2. The median (interquartile range) POD for initiating positioning techniques post-CEA application was POD 0 (0,0); POD 5 (3,7) for active and passive elongation; POD 12 (4.25,14.25) for out-of-bed mobilization and functional mobility training; and POD 22.5 (8,25) for return to treatment in burn therapy clinic. Additionally, Figure 1A demonstrates the actual POD resumption of elongation techniques, out-of-bed mobilization, and treatment in a burn therapy clinic for each study patient chronologically. Related, Figure 2A represents the resumption of each of these interventions by year over the study time period.

**Table 2.** Post-CEA Therapy Resumption

Intervention	POD^a^
Positioning	0 (0,0)
Elongation	5 (3,7)
Out-of-bed mobilization	12 (4.25,14.25)
Out-of-room treatment resumed in the therapy clinic	22.5 (8,25)

^a^POD presented as median (interquartile range).

Abbreviations: CEA, cultured epidermal autografts; POD, postoperative day.

Discharge disposition is depicted in Table 3. Eight patients were admitted to the burn-specific inpatient rehabilitation facility (IRF), one patient was transferred to a skilled nursing facility, and one patient was discharged home. Table 4 consists of patient outcomes that were recorded at the time of discharge. Eight patients were independent in functional status at the time of their discharge from the burn center, and returned to preburn activities. None of the 10 patients demonstrated the presence of a joint contracture upon their discharge, and at 6 months postdischarge from the burn center, none of the 10 study patients had required additional surgery or reconstructive procedure. The mean CEA-graft take at discharge among all 10 patients was 95.5% ± 3.95%.

**Table 3.** Discharge Disposition

Discharge placement	Number of patients
Burn IRF	8
SNF	1
Home	1

Abbreviations: IRF, inpatient rehabilitation facility; SNF, skilled nursing facility.

**Table 4.** Outcomes at Time of Discharge

	Patients^a^
Independent functional status at discharge to home	8 (80)
Return to preburn activities	8 (80)
One or more joint contractures present	0 (0)
Contracture release procedures required up to 6 months postdischarge	0 (0)
Average CEA-graft take at discharge^b^	96% ± 3.95%

^a^ *n* (%).

^b^Mean ± standard deviation.

## DISCUSSION

Early mobility of burn patients, before and after standard skin grafting procedures, is widely utilized as the gold standard across burn centers, as research continues to demonstrate the direct and positive effects that early therapy intervention has on patient outcomes.^6^ However, early mobilization in CEA-treated patients is not considered standard practice for all burn centers, likely due to the lack of research available regarding therapy intervention in this specialized population. Some barriers may also include inexperience among burn staff in the treatment of patients post-CEA, high potential for shear secondary to the physiological fragility of CEA, the increased cost of this particular procedure, and understaffing. Despite all of this, early mobilization of burn patients, following surgical application of CEA, can be accomplished without harm to CEA sites through the effective execution of a therapy-centric post-CEA treatment plan by an educated burn therapy team.

The burn rehabilitation guidelines utilized by this center, summarized in Table 5, were developed in an attempt to lessen the negative physiological outcomes associated with delayed mobilization and prolonged bedrest that has been reported in patients following CEA application.^3^ As demonstrated in this retrospective case series, early implementation of these guidelines by appropriately trained burn therapists resulted in optimal mobility outcomes at discharge, including a 0% contracture rate and 80% of patients returning to preburn activity level. Furthermore, it was determined that all 10 patients had greater than 90% CEA-graft take at discharge, with 0% CEA-graft loss related to a patient’s therapy treatment. Although the majority of patients in this study had poor long-term follow-up after discharge, 0% of patients had required an invasive, reconstructive surgical procedure 6 months following their discharge from the burn center.

**Table 5.** Burn Rehabilitation Guidelines

Pre-CEA (24-72 h of admission) The patient was identified as a CEA candidate. PT/OT evaluations completed. OR for BWE + allograft or skin substitute. The skin sample was harvested and sent for CEA growth.
Pre-CEA (3- to 4-week period) Daily hydrotherapy and burn wound assessment to decrease the risk of infection. OR for re-excision to decrease necrotic tissue as needed. Therapy intervention is extremely important (2 sessions per day): Elongation + positioning interventions to maintain adequate motion. Muscular strength + endurance training.
Post-CEA (POD 0-1) Immediate therapy intervention: Positioning to decrease pressure on CEA. PT/OT assistance in all patient movements to decrease shear. Complex wound care, bilirubin light treatment.
Post-CEA (POD 2-6) Elongation/ROM (2+ h/day): Requires at least 2 therapists to be present. Out-of-bed mobilization: Initiated with MD approval.
Post-CEA (7-10+) Resume treatment in the burn therapy clinic. 2-5 h intensive therapy session (5-7 days/week). Discharge to the next level of care or home. Admission to inpatient burn rehabilitation (IRF).

Abbreviations: BWE, burn wound excision; CEA, cultured epidermal autografts; OR, operating room; ROM, range of motion.

Subsequently, there has been a notable decrease in length of stay (LOS) in the acute care and IRF settings when comparing CEA-treated patients in 2015 to patients treated post-CEA application in 2023, as seen in Figure 2. This decline in LOS could potentially be a result of earlier implementation of therapy interventions with each consecutive CEA-treated patient due to increasing staff comfort levels and experience. Fortunately, the burn rehabilitation staff at this center suffered from no attrition during this time period.

In conclusion, an intensive burn rehabilitation program should not be controversial in burn patients after the CEA procedure. Severely burned patients treated with CEA can be mobilized early without adverse effects on CEA sites if there exists a team of dedicated burn therapists properly trained in a post-CEA therapy treatment plan or burn rehabilitation guidelines, implemented postapplication immediately to enhance patient outcomes related to overall functional status and quality of life.

## Notes

**Author Contributions:** Sarah E. Sabbatini (Conceptualization [lead], Data curation [lead], Formal analysis [lead], Investigation [lead], Methodology [lead], Writing—original draft [lead]), David M. Hill (Resources [supporting], Writing—review & editing [supporting]), and Sai R. Velamuri (Supervision [supporting], Writing—review & editing [supporting])

**Funding:** This research did not receive any specific grant from funding agencies in the public, commercial, or not-for-profit sectors. Vericel supplied nonrestrictive educational grant funds to Regional One Health’s Foundation for support for travel and will provide the Regional One Health Foundation with a grant for open access licensure pending publication.
